# Identification of Simplified Microbial Communities That Inhibit Clostridioides difficile Infection through Dilution/Extinction

**DOI:** 10.1128/mSphere.00387-20

**Published:** 2020-07-29

**Authors:** Jennifer M. Auchtung, Eva C. Preisner, James Collins, Armando I. Lerma, Robert A. Britton

**Affiliations:** a Alkek Center for Metagenomics and Microbiome Research, Baylor College of Medicine, Houston, Texas, USA; b Department of Molecular Virology and Microbiology, Baylor College of Medicine, Houston, Texas, USA; c Nebraska Foods for Health Center, University of Nebraska—Lincoln, Lincoln, Nebraska, USA; d Department of Food Science and Technology, University of Nebraska—Lincoln, Lincoln, Nebraska, USA; University of Michigan—Ann Arbor

**Keywords:** *Clostridioides difficile*, FMT, colonization resistance, microbiome, simplified communities

## Abstract

The gastrointestinal microbiome plays an important role in limiting susceptibility to infection with Clostridioides difficile. To better understand the ecology of bacteria important for C. difficile colonization resistance, we developed an experimental platform to simplify complex communities of fecal bacteria through dilution and rapidly screen for their ability to resist C. difficile colonization after challenge, as measured by >100-fold reduction in levels of C. difficile in challenged communities. We screened 76 simplified communities diluted from cultures of six fecal donors and identified 24 simplified communities that inhibited C. difficile colonization *in vitro*. Sequencing revealed that simplified communities were composed of 19 to 67 operational taxonomic units (OTUs) and could be partitioned into four distinct community types. One simplified community could be further simplified from 56 to 28 OTUs through dilution and retain the ability to inhibit C. difficile. We tested the efficacy of seven simplified communities in a humanized microbiota mouse model. We found that four communities were able to significantly reduce the severity of the initial C. difficile infection and limit susceptibility to disease relapse. Analysis of fecal microbiomes from treated mice demonstrated that simplified communities accelerated recovery of indigenous bacteria and led to stable engraftment of 19 to 22 OTUs from simplified communities. Overall, the insights gained through the identification and characterization of these simplified communities increase our understanding of the microbial dynamics of C. difficile infection and recovery.

**IMPORTANCE** Clostridioides difficile is the leading cause of antibiotic-associated diarrhea and a significant health care burden. Fecal microbiota transplantation is highly effective at treating recurrent C. difficile disease; however, uncertainties about the undefined composition of fecal material and potential long-term unintended health consequences remain. These concerns have motivated studies to identify new communities of microbes with a simpler composition that will be effective at treating disease. This work describes a platform for rapidly identifying and screening new simplified communities for efficacy in treating C. difficile infection. Four new simplified communities of microbes with potential for development of new therapies to treat C. difficile disease are identified. While this platform was developed and validated to model infection with C. difficile, the underlying principles described in the paper could be easily modified to develop therapeutics to treat other gastrointestinal diseases.

## Abstract (precis)

Clostridioides difficile is the leading cause of antibiotic-associated diarrhea and a significant health care burden. Fecal microbiota transplantation is highly effective at treating recurrent C. difficile disease; however, uncertainties about the undefined composition of fecal material and potential long-term unintended health consequences remain. These concerns have motivated studies to identify new communities of microbes with a simpler composition that will be effective at treating disease. This work describes a platform for rapidly identifying and screening new simplified communities for efficacy in treating C. difficile infection. Four new simplified communities of microbes with potential for development of new therapies to treat C. difficile disease are identified. While this platform was developed and validated to model infection with C. difficile, the underlying principles described in the paper could be easily modified to develop therapeutics to treat other gastrointestinal diseases.

## INTRODUCTION

Clostridioides difficile is the most common cause of antibiotic-associated diarrhea (1–3). Estimates of annual health care costs associated with treating C. difficile infection (CDI) in the United States range from $1 billion to $4.8 billion (4). Although uncomplicated infections are typically self-limiting, severe infections require treatment (5). For approximately 25% of patients, resolution of primary infection is followed by one or more rounds of recurrent C. difficile infection (rCDI) (6), which diminishes the quality of life and contributes to overall health care costs (7, 8).

To cause disease, ingested C. difficile spores must germinate into vegetative cells that produce toxins. The bile salt cholate and its derivatives stimulate C. difficile germination, along with cogerminants glycine and other amino acids (9, 10). The gastrointestinal microbiome plays key roles in limiting symptomatic CDI by competing with C. difficile for nutrients (11–13), producing metabolites that inhibit C. difficile growth (14–18), maintaining immune homeostasis (19–21), and metabolizing primary bile salts into secondary bile salts that inhibit the growth of vegetative cells (9, 22, 23). Antibiotic treatment leads to loss of GI microbiome diversity (24–26), is a key risk factor for primary infection (27–30), and contributes to susceptibility to recurrent disease.

Several different therapies are currently used to treat rCDI and act to limit different aspects of cell growth and pathogenicity. Clinical cure rates of 70% have been reported following extended-pulsed administration of the antibiotic fidaxomicin (31). An 80% cure rate has been reported for patients with primary and recurrent CDI treated with bezlotoxumab, an antibody that targets C. difficile toxin B (32). Fecal microbiome transplantation (FMT) for treatment of rCDI has reported cure rates from 44 to 100% (33, 34). However, concern for potential adverse events (for examples, see reference 35) motivates ongoing studies to develop alternatives to FMT to treat rCDI.

Defined community microbial therapeutics are one alternative to FMT under investigation. Previous studies have demonstrated success in administration of a consortium of 10 (36) or 33 (37) human fecal bacteria for treatment of rCDI. Despite these advances, no defined microbial therapeutic is currently available for treatment of CDI. One limitation to developing microbial community therapeutics is the availability of appropriate models for rapid screening. We developed a coupled *in vitro* and *in vivo* platform to screen simple microbial communities for their ability to prevent CDI. We identified four simplified communities that limited C. difficile growth *in vitro* and reduced the severity of disease *in vivo*. While the potential of these communities to treat disease in humans is unknown, the approaches could be applied to identification of additional simplified communities to treat rCDI and microbiome-linked diseases.

## RESULTS

### Identification of simplified communities that limit C. difficile growth *in vitro*.

**FIG 1:**
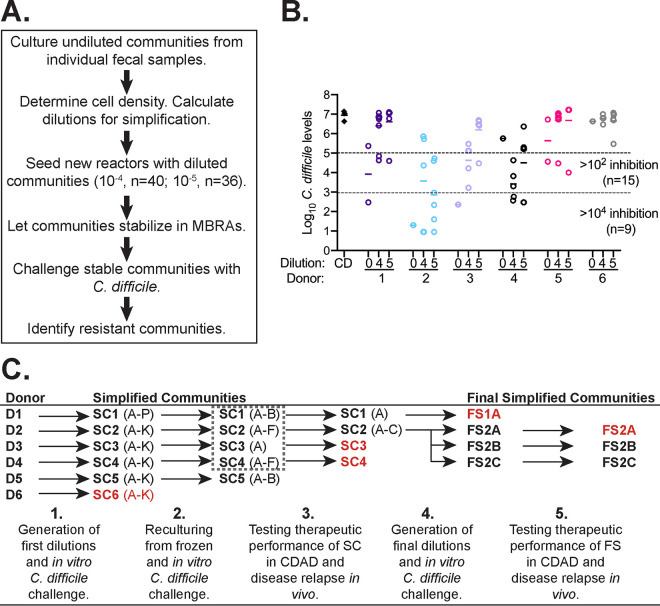
Identification of simplified communities that inhibit C. difficile proliferation through dilution/extinction community assembly. (A) Overview of process to identify simplified communities. (B) Log_10_ C. difficile levels measured in diluted communities on day 5 or 6 following challenge. Closed triangles, C. difficile (CD) cultured alone; open circles:, C. difficile cultured in the presence of undiluted communities (0) and communities diluted 10^−4^ (4) and 10^−5^ (5) cultured from indicated fecal sample donors. Solid lines represent geometric means of samples. Dashed lines demarcate inhibition of C. difficile levels by 10^2^ and 10^4^, with the number of samples per category indicated. (C) Schematic representation of samples generated over the course of experiments as described in the text. Red typeface indicates samples that failed to provide protection from C. difficile colonization at the step indicated.

To identify simplified communities that could suppress C. difficile, we applied a dilution/extinction strategy (38, 39) as outlined in Fig. 1A. In this approach, undiluted fecal communities are simplified through dilution, with abundant organisms preserved and rare organisms randomly distributed or lost as predicted by the Poisson distribution.

Undiluted fecal communities were established in minibioreactor arrays (MBRAs) from six individual fecal donors (D1 to D6) and allowed to stabilize (Fig. 1A). Cell density was measured by nonselective plating and used with published operational taxonomic unit (OTU) abundance data (40) to estimate dilutions needed to simplify communities. Following dilution, simplified communities were stabilized in continuous culture before challenge with 10^4^ vegetative C. difficile cells. We measured C. difficile proliferation in each bioreactor over time and identified nine simplified communities that lowered C. difficile levels >10,000 times and 15 communities that decreased C. difficile levels >100 times compared to C. difficile cultivated alone in bioreactors (Fig. 1B).

### CDI-resistant simplified communities separate into distinct community types.

**FIG 2:**
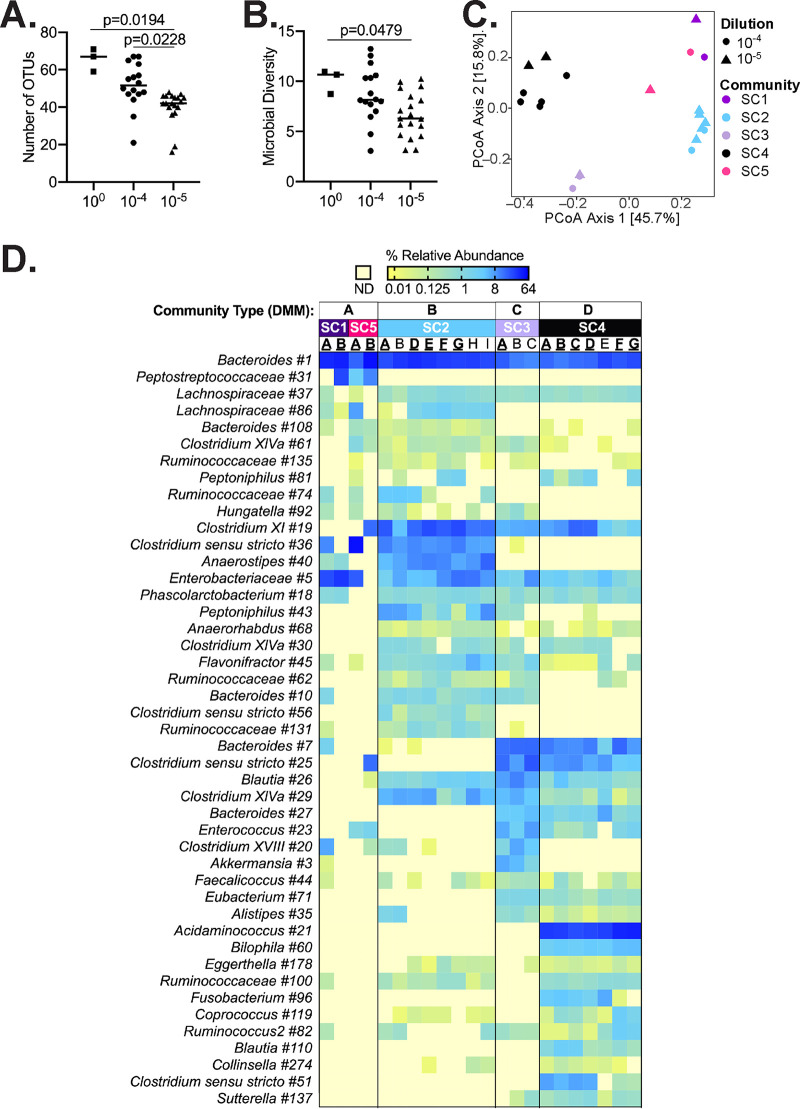
Comparison of microbial communities present in undiluted and simplified communities that inhibit C. difficile growth. (A and B) The number of OTUs (A) and microbial diversity (inverse Simpson) (B) detected in undiluted communities (squares) and communities diluted 10^−4^ (circles) and 10^−5^ (triangles) are plotted. Lines represent medians; *P* values of <0.05 as calculated by one-way Kruskal-Wallis testing are reported. (C) Principal-coordinate analysis (PCoA) visualization of Bray-Curtis dissimilarities between C. difficile-resistant communities SC1 to SC5. Colors represent different SCs as indicated; dilutions are indicated by shapes. Percent variation described by each axis is indicated in parentheses. (D) Forty-five OTUs that most significantly contribute to partitioning of SC1 to SC5 into the indicated four community types (A to D). The log_2_-transformed percent abundance of each OTU is plotted across all samples. Samples are arranged by donor and community type as indicated at the top. Values ranged from 0.01% (yellow) to 64% (dark blue) of total sequences as indicated; pale yellow indicates that there were no detected sequences (ND). Simplified community replicate names are indicated in boldface, underlining indicates communities selected for reculturing as described in the text. Table S1 contains data from all 147 OTUs that account for differences between community types.

To better understand how dilution impacted community composition, we compared the 16S rRNA gene content between a subset of undiluted donor communities (D2 to D4) and C. difficile*-*resistant simplified communities (SC1 to SC5) (Fig. 1C). The median number of OTUs in the undiluted communities was 67; dilution reduced the median numbers of OTUs to 50 and 42 in the simplified communities diluted 10^−4^ and 10^−5^, respectively (Fig. 2A).

10.1128/mSphere.00387-20.5 TABLE S1 OTUs that contribute to partitioning of simplified communities by Dirichlet multinomial mixtures. Download Table S1, XLSX file, 0.04 MB. Copyright © 2020 Auchtung et al. 2020 Auchtung et al. This content is distributed under the terms of the Creative Commons Attribution 4.0 International license.

We compared differences in community structure across simplified communities (Fig. 2C) and used Dirichlet multinomial mixtures to identify community types and discriminatory taxa (41) (Fig. 2D). We found that the data best partitioned into four community types, with community type A containing SC1 and SC5 communities, while SC2, SC3, and SC4 separated into community types B to D, respectively.

Forty-five OTUs accounted for 80% of the difference between community types in the model (Fig. 2D). Many of these OTUs were present in all samples, but they varied in abundance by community type. Notable features of community type A included *Bacteroides*, *Peptostreptococcaceae*, and *Lachnospiraceae* OTUs. *Peptostreptococcaceae* number 31, which was detected in 3 of 4 type A communities, was 100% identical to C. difficile 16S rRNA, indicating potential experimental contamination of these communities prior to C. difficile challenge. Type B communities had higher levels of *Anaerostipes*, *Clostridium_sensu_stricto*, *Clostridium* XI, and *Peptoniphilus*; *Akkermansia*, *Blautia*, *Clostridium* XVIII, and *Enterococcus* were enriched in type C communities; and *Acidaminococcus*, *Bilophila*, and *Fusobacterium* were enriched in type D communities.

### Simplified communities retain the ability to suppress C. difficile when recultured.

We selected 17 of the 24 C. difficile-resistant SC1 to SC5 cultures for retesting based on previous ability to suppress C. difficile growth (9/9 that suppressed >10^4^; 8/24 that suppressed >10^2^) and variation in microbiome composition (indicated by boldface type in Fig. 2D). Simplified communities were established from frozen stocks in triplicate MBRAs and allowed stabilization prior to challenge with C. difficile. Thirteen of the 17 communities were able to inhibit C. difficile upon reculturing (Fig. 1C; see also Fig. S1 in the supplemental material). Five communities suppressed C. difficile growth >10,000-fold across all three replicates, two suppressed C. difficile >100-fold across all three replicates, and six communities suppressed C. difficile in at least one replicate (Fig. S1). We selected one replicate from each community type described in Fig. 2D that was also able to suppress C. difficile upon reculturing to test in a mouse model of disease. SC1A, SC2A, SC3A, and SC4A replicates were selected; replicate names were simplified to SC1 to SC4 for testing in mice.

10.1128/mSphere.00387-20.1 FIG S1 C. difficile proliferation in recultured triplicate cultures seeded with initially suppressive communities. C. difficile levels from triplicate reactors (closed circles) recultured from cryopreserved simplified communities are plotted with the simplified community designation and replicate letter indicated below the graph as indicated in Fig. 2 in the main text. Open circles indicate levels of C. difficile detected in the initial culture reported in Fig. 1B in the main text. Lines represent medians of all four data points. Dotted lines indicate levels of C. difficile that are >10^2^ and >10^4^ times lower than the maximum C. difficile levels reported in Fig. 1. Download FIG S1, PDF file, 0.4 MB. Copyright © 2020 Auchtung et al. 2020 Auchtung et al. This content is distributed under the terms of the Creative Commons Attribution 4.0 International license.

### SC1 and SC2 suppress CDAD.

**FIG 3:**
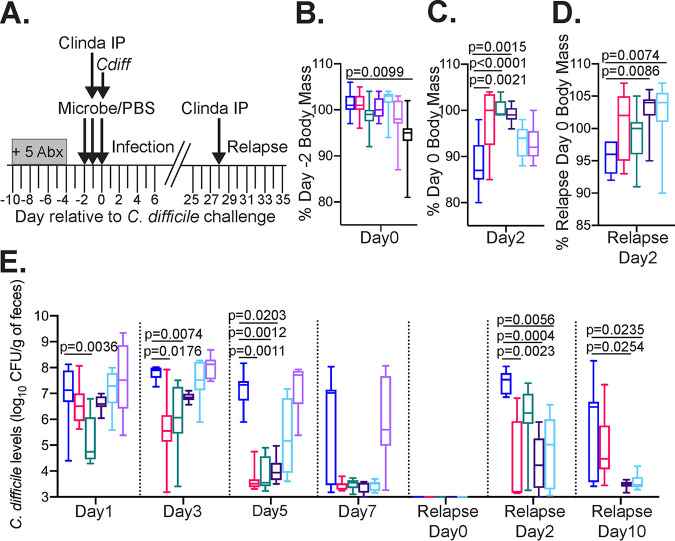
SC1 and SC2 suppress C. difficile*-*associated disease in the ^HMb^mouse model. (A) Overview of infection and recurrence protocol used to evaluate simplified communities and FMT treatments. Mice were treated with a mixture of five antibiotics (+5 Abx) in the drinking water for 6 days as described previously (42). After 2 days on fresh water, mice were treated once daily with microbial communities as indicated in panels B to E or with a vehicle (PBS) for 3 days. One day prior to C. difficile infection, clindamycin was administered intraperitoneally (Clinda IP). On the third day of administration of microbial communities, mice were challenged with C. difficile (*Cdiff*). Disease severity and levels of C. difficile were monitored during initial infection and following induction of relapse with intraperitoneal injection of clindamycin. In panels B to E, treatments are indicated as follows: PBS, dark blue (*n* = 9); ^HMb^mouse FMT (M-FMT), magenta (*n* = 9); human FMT (H-FMT), teal (*n* = 9); SC1, dark purple (*n* = 9); SC2, light blue (*n* = 9); SC3, violet (*n* = 9); and SC4, black (*n* = 8). (B) Percentage of day −2 body mass on day 0 (prior to C. difficile challenge) following 2 days of treatment with simplified communities. (C) Percentage of day 0 body mass on day 2 following initial infection (D) Percentage of relapse day 0 body mass 2 days following relapse. (E) Levels of C. difficile measured in the feces of treated mice on days indicated below the graph. Boxes represent the interquartile ranges, horizontal lines indicate the medians, and vertical lines indicate the ranges of data collected from replicate mouse samples at each time point. Significance of differences between microbe- and PBS-treated mice in each panel were evaluated with one-way Kruskal-Wallis testing with Dunn’s correction for multiple comparisons. *P* values less than 0.05 are reported. Two mice lost from PBS (days 3 and 4) and ^HMb^mouse FMT-treated groups (day 3) were included in calculations until death. Mice treated with SC3 were not tested for resistance to recurrent infection. C. difficile levels in H-FMT-treated mice were not tested on relapse day 10. Longitudinal data collected during initial infection and relapse are plotted in Fig. S2.

We tested SC1 to SC4 for the ability to suppress C. difficile-associated disease (CDAD) in a humanized microbiota mouse (^HMb^mouse) model of disease (42). Two positive controls were used to test for suppression of CDAD: FMT freshly prepared from our colony of ^HMb^mice (M-FMT) and a cryopreserved aliquot of human FMT previously used successfully in a CDI fecal transplant program (H-FMT; T. Savidge, personal communication). Mice were treated with antibiotics to disrupt the microbiome and then gavaged with cells from simplified communities SC1 to SC4, M-FMT, or H-FMT on 3 consecutive days; control mice were treated with a vehicle (phosphate-buffered saline [PBS]) (Fig. 3A). Body mass was measured daily beginning with the first day of gavage to test for potential toxicity of communities. Because mice treated with SC4 exhibited ∼5% body mass loss from baseline prior to C. difficile challenge (Fig. 3B), they were excluded from further analyses.

10.1128/mSphere.00387-20.2 FIG S2 Simplified microbial communities SC1 and SC2 suppress C. difficile in ^HMb^mouse model of CDI. Longitudinal data collected from ^HMb^mice that were administered treatments described in Fig. 3 in the main text. (A to I) Percentage of day 0 body mass (A to D) and Log_10_ C. difficile levels in feces (E to I) of mice over the first 7 days following infection. (L to R) Percentage of relapse day 0 body mass (L to O) and log_10_ C. difficile levels in feces (P to R) of mice over time following initiation of relapse by intraperitoneal (i.p.) injection of clindamycin. Data presented are from mice treated with ^HMb^mouse FMT (M-FMT [A, F, K, and O]), human FMT (H-FMT [B, G, L, and P]), SC1 (C, H, M, and Q), SC2 (D, I, N, and R) and SC3 (E and J). Data from PBS-treated mice (dark blue lines) are repeated in each panel for reference. Lines indicate medians and error bars indicate interquartile ranges. Data points identified as statistically significant in Fig. 3 are indicated by asterisks. Response during relapse was not tested in SC3-treated mice. Download FIG S2, PDF file, 1.7 MB. Copyright © 2020 Auchtung et al. 2020 Auchtung et al. This content is distributed under the terms of the Creative Commons Attribution 4.0 International license.

Following C. difficile challenge, mice treated with PBS exhibited a decline in body mass (Fig. 3C) and shed C. difficile in their feces (Fig. 3E). In contrast, SC1-treated mice maintained their body mass, with levels similar to those observed in the two positive controls, M-FMT- and H-FMT-treated mice (Fig. 3C), and exhibited more rapid clearance of C. difficile in feces (Fig. 3E). Trends toward reduced body mass loss in SC2- and SC3-treated mice (Fig. 3C) and more rapid clearance of C. difficile in feces of SC2-treated mice (Fig. 3E) were not statistically significant.

Because C. difficile was cleared more rapidly in SC1- and SC2-treated mice, we tested whether these communities would reduce susceptibility to recurrent disease. Previously, we demonstrated that relapse could be induced through a single intraperitoneal (i.p.) injection of clindamycin (42). Four weeks following the initial C. difficile challenge, the majority of mice no longer shed C. difficile at detectable levels in their feces (Fig. 3E). We treated mice with a single clindamycin i.p. injection and then measured changes in body mass and C. difficile levels. Consistent with relapse, we observed a modest body mass decline (Fig. 3D) and an increase of C. difficile in feces (Fig. 3E) of PBS-treated mice. In contrast, there was a modest body mass gain, reduced shedding of C. difficile in feces, and more rapid clearance of C. difficile in SC1- and SC2-treated mice following relapse. M-FMT- and H-FMT-treated mice had a more modest body mass gain that was not statistically significant from the mass of PBS-treated mice.

### SC2 can be further simplified and still inhibit C. difficile growth.

**FIG 4:**
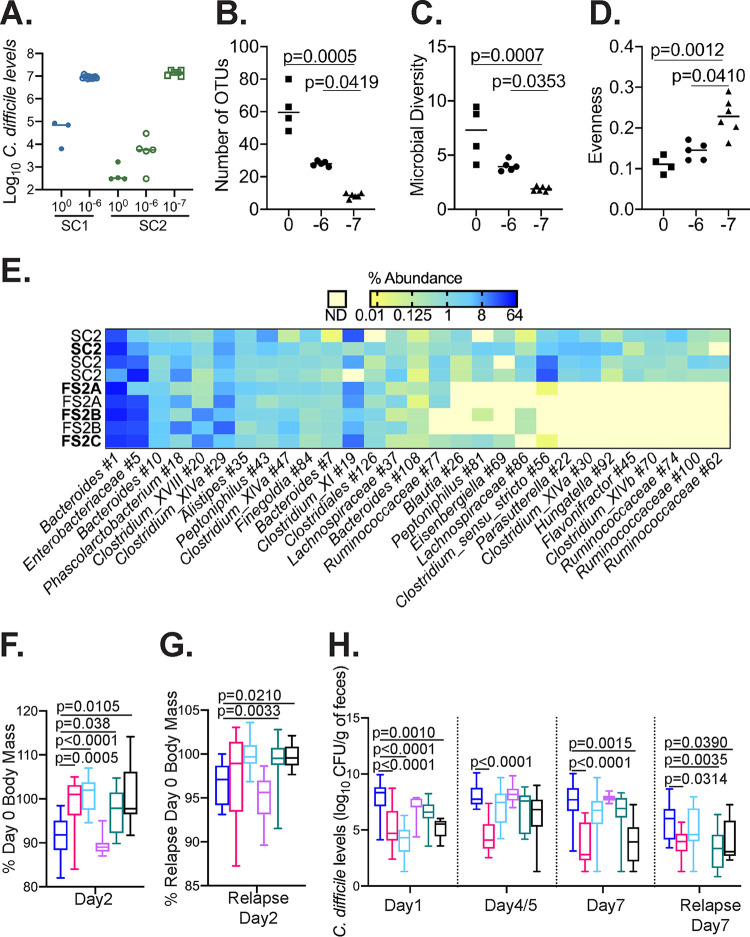
Identification of final simplified (FS) microbial communities that suppress C. difficile in MBRA and ^HMb^mouse models of CDI. (A) Plot of log_10_ C. difficile levels on the final day in culture with recultured SC1 (closed blue circles) and SC2 (closed green circles) and in recultured SC1 and SC2 that were further simplified to 10^−6^-fold (open circles) and 10^−7^-fold (open squares). Lines represent medians. (B to D) Number of OTUs (B), microbial diversity (inverse Simpson index) (C), and species evenness (Simpson evenness index) (D) of recultured SC2 and communities diluted 10^−6^ and 10^−7^. Lines represent medians; any significant differences (*P* < 0.05) detected in distributions of communities as determined by one-way Kruskal-Wallis testing are reported. (E) Differences in abundance of OTUs present above 0.1% of total sequences in at least two replicate SC2, FS2A, FS2B, or FS2C cultures. Samples are indicated to the left of the plot; data in boldface type are from the cultures shown in panel A; the first SC2 replicate is from the data reported in Fig. 2, and the additional SC2, FS2A, and FS2B replicates were collected from bioreactor cultures used to gavage ^HMb^mice in panels F to H. Yellow represents <0.01% abundance and blue represents > 64% of total sequences as indicated by shading of log_2_-transformed relative abundance data; pale yellow indicates that no sequences were detected (ND). In panels F to H, data were collected from ^HMb^mice subjected to treatment indicated as follows: PBS, dark blue (*n* = 18); M-FMT, magenta (*n* = 17); SC2, light blue (*n* = 17); FS2A, violet (*n* = 9); FS2B, teal (*n* = 17); and FS2C, black (*n* = 8). Treatments were administered as described for Fig. 3A. (F) Percentage of day 0 body mass on day 2 of infection. (G) Percentage of relapse day 0 body mass on day 2 of relapse. (H) Log_10_ levels of C. difficile in mouse feces on days indicated. Boxes represent the interquartile ranges, horizontal lines indicate the medians, and vertical lines indicate the ranges of data collected from replicate mouse samples at each time point. Significant differences (*P* < 0.05) in distributions of community-treated mice compared to PBS-treated mice as determined by one-way Kruskal-Wallis testing with Dunn’s correction for multiple comparisons are reported. Longitudinal data from treatments shown in panels F to H are reported in Fig. S3.

To test whether either SC1 or SC2 could be further simplified, cultures were diluted to a concentration of 250 CFU/ml (10^−6^ dilution); Poisson calculations indicated that this dilution should reduce the complexities of SC1 and SC2 to 17 and 31 OTUs, respectively. Final simplified communities (FS) were allowed to stabilize prior to challenge with C. difficile. Although 10^−6^ dilutions of SC1 (designated FS1A to -E) lost the ability to inhibit C. difficile, 10^−6^ dilutions of SC2 (designated FS2A to -E) continued to inhibit C. difficile growth (Fig. 4A). C. difficile inhibition was lost when SC2 communities were diluted another 10-fold.

10.1128/mSphere.00387-20.3 FIG S3 FS2B and FS2C communities suppress C. difficile in the ^HMb^mouse model of CDI. Longitudinal data collected from ^HMb^mice that were administered treatments as for Fig. 4 and as described in the main text are shown. (A to J) Percentage of day 0 body mass (A to E) and log_10_ levels of C. difficile in feces over time (F to J) during the first 7 days following infection. (K to S) Percentage of relapse day 0 body mass (K to O) and levels of C. difficile in feces over time (P to S) during the 7 days following initiation of relapse by i.p. injection of clindamycin. Data are from mice treated with M-FMT (A, F, K, and P), SC2 (B, G, L, and Q), FS2A (C, H, and M), FS2B (D, I, N, and R) and FS2C (E, J, O, and S). Data from PBS-treated mice (dark blue lines) are repeated in each panel for reference. Lines represent median values, and error bars represent interquartile ranges. Data points identified as statistically significant in Fig. 4 are indicated by asterisks. Download FIG S3, PDF file, 1.2 MB. Copyright © 2020 Auchtung et al. 2020 Auchtung et al. This content is distributed under the terms of the Creative Commons Attribution 4.0 International license.

We analyzed the effects of further simplification on community composition and found that the number of OTUs declined from a median of 60 in SC2 cultures to medians of 28 and 9 in communities diluted 10^−6^ and 10^−7^ (Fig. 4B). In addition, microbial diversity decreased (Fig. 4C) and species evenness increased (Fig. 4D) with increasing levels of dilution. The majority of OTUs lost through dilution were classified in the order *Clostridiales* (Fig. 4E). We selected three final simplified communities (FS2A, FS2B, and FS2C) to test for the ability to inhibit CDAD in ^HMb^mice.

### FS2B and FS2C suppress CDAD in ^HMb^mice.

We used the experimental approach outlined in Fig. 3 to test final simplified communities FS2A, FS2B, and FS2C. As controls, we administered M-FMT, SC2, and PBS. Similar to the findings in our initial study, PBS-treated mice exhibited body mass loss following challenge with C. difficile and weight loss was prevented by treatment with M-FMT (Fig. 4F). Treatment with SC2 prevented body mass loss, contrasting with prior results in which SC2 treatment was partially protective. Changes in efficacy could be due to the shifts in microbial composition upon the reculturing of SC2 (Fig. 4E). FS2B- and FS2C-treated mice were also protected from CDAD, whereas mice treated with community FS2A lost body mass at a level similar to that of PBS-treated mice (Fig. 4F).

PBS-treated mice shed C. difficile in feces at similarly high levels across all time points tested (Fig. 4H). In FS2C-treated mice, C. difficile levels were significantly lower than in PBS-treated mice on day 1 and day 7 of infection. In SC2-treated mice, C. difficile levels were significantly lower than in PBS-treated mice only on day 1 of infection. FS2A- and FS2B-treated mice showed little reduction in levels of C. difficile shedding. M-FMT-treated mice had significantly lower levels of C. difficile than PBS-treated mice at all time points.

Following induction of relapse, we observed an ∼3% median reduction in body mass in PBS-treated mice on day 2 following i.p. injection (Fig. 4G). Mice treated with SC2, FS2B, or FS2C showed <0.5% median body mass loss, whereas FS2A-treated mice exhibited ∼5% median body mass loss. M-FMT-treated mice exhibited ∼1% decrease in body mass. We also observed more rapid clearance of C. difficile in FS2B- and FS2C-treated mice (Fig. 4H).

### Treatment with simplified communities has persistent effects on the fecal microbiome.

We analyzed microbial communities from mouse fecal samples on days 1, 4 or 5, and 7 and relapse days 0, 2, and 7. Sequence data obtained from mice were pooled with bioreactor data to facilitate tracking of bacteria present in simplified communities in treated mice. We found that sequences that clustered at >99% average nucleotide identity (ANI) (Table S2) provided greater resolution of OTUs distinct to *in vitro*-cultured simplified communities and M-FMT-treated ^HMb^mice than sequences that clustered at >97% ANI (Table S3). With sequences clustered at >99% ANI, 90% of OTUs found in M-FMT-treated ^HMb^mice were not detected in *in vitro* cultures and likely represent indigenous bacteria. Similarly, 81% of OTUs found in *in vitro* cultures were not detected in FMT-treated ^HMb^mice. Subsequent analyses used OTUs that clustered at >99% ANI.

10.1128/mSphere.00387-20.6 TABLE S2 Characterization of OTUs clustered at >99% ANI shared by simplified communities and ^HMb^mice. Download Table S2, XLSX file, 0.03 MB. Copyright © 2020 Auchtung et al. 2020 Auchtung et al. This content is distributed under the terms of the Creative Commons Attribution 4.0 International license.

10.1128/mSphere.00387-20.7 TABLE S3 Characterization of OTUs clustered at >97% ANI shared by simplified communities and ^HMb^mice. Download Table S3, XLSX file, 0.02 MB. Copyright © 2020 Auchtung et al. 2020 Auchtung et al. This content is distributed under the terms of the Creative Commons Attribution 4.0 International license.

**FIG 5:**
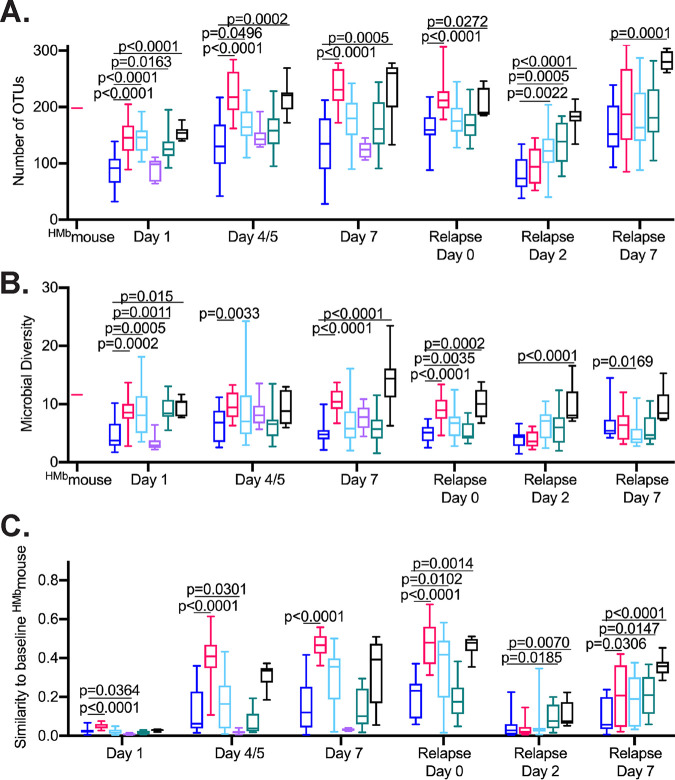
Treatment with SC2 and FS2C restores microbial diversity and shifts microbiome composition toward baseline state observed in ^HMb^mice not treated with antibiotics. 16S rRNA gene sequence data were obtained from bacteria present in the feces of mice treated with PBS (dark blue), M-FMT (magenta), SC2 (light blue), FS2A (violet), FS2B (teal), or FS2C (black) on days 1, 4 or 5, and 7 following initial C. difficile infection and days 0, 2, and 7 relative to initiation of relapse with clindamycin i.p. (For FS2A-treated mice, 16S rRNA gene sequence data were obtained only from samples collected during initial infection for FS2A-treated mice.) 16S rRNA sequence data were also obtained from a pooled fecal sample collected from an ^HMb^mice not treated with antibiotics that was used for FMT administration. (A and B) Number of OTUs (A) and microbial diversity (inverse Simpson index) (B) measured in sample collected from ^HMb^mice not treated with antibiotics used for FMT administration (^HMb^mouse) and in samples collected from treated mice at time points indicated below the graph. (C) Similarity to baseline ^HMb^mouse sample used for FMT administration measured in samples at time points indicated below graph (similarity = 1 − Bray-Curtis dissimilarity). Boxes represent the interquartile ranges, horizontal lines indicate the medians, and vertical lines indicate the ranges of data collected from replicate mouse samples at each time point. Significance of differences in microbe-treated compared to PBS-treated animals at each time point were evaluated with one-way Kruskal-Wallis testing with Dunn’s correction for multiple comparisons; *P* values of <0.05 are reported.

The number of OTUs detected on day 1 following infection were 25 to 50% lower across all treatment groups than those observed in the FMT sample collected from mice not treated with antibiotics (Fig. 5A, ^HMb^mouse). Treatment with M-FMT partially restored OTU abundance and microbial diversity on day 1; full recovery to levels observed in untreated mice was not observed until day 4 or 5 during infection. In PBS-treated mice, the median number of OTUs detected in fecal samples increased over time but did not return to the levels detected in untreated mice. Treatment with SC2, FS2C, or FS2B significantly increased the number of OTUs detected on day 1 compared to that in PBS-treated mice. Later increases in OTU abundance in FS2C-treated mice paralleled findings for FMT-treated mice. For SC2- and FS2B-treated mice, OTU abundance increased over time but not to the extent observed in FMT-treated mice. Neither OTU abundance nor microbial diversity was significantly different between FS2A-treated and PBS-treated mice over the first week of infection. Treatment with FMT, SC2, FS2B, and FS2C also significantly increased microbial diversity compared to that in PBS-treated mice (Fig. 5B).

Similarities in community composition between the baseline ^HMb^mouse sample not treated with antibiotics and communities in the feces of treated mice were calculated (Fig. 5C). In M-FMT-treated mice, fecal communities had low similarities to the baseline ^HMb^mouse sample on day 1, but similarities increased by day 7 (Fig. 5C). In contrast, similarities between PBS-treated mice and the baseline untreated ^HMb^mouse sample were significantly lower than M-FMT-treated mice through relapse day 0. Compared to PBS-treated mice, FS2C- and SC2-treated mice exhibited an accelerated return toward the baseline microbiome composition. FS2B-treated mice exhibited a return to baseline microbiome that paralleled that in PBS-treated mice. In contrast, FS2A-treated mice exhibited significantly reduced recovery of microbiome composition compared to that of PBS-treated mice, indicating that FS2A treatment may suppress recovery of the fecal microbiome.

### Treatments shift the composition of indigenous bacteria.

**FIG 6:**
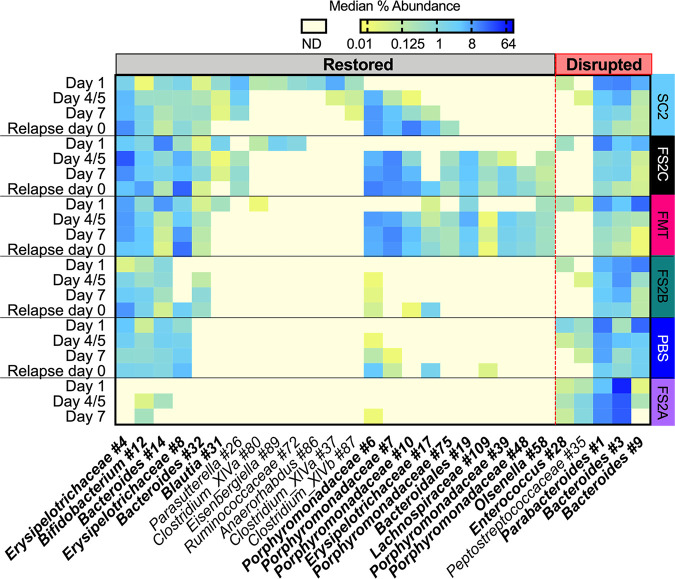
Treatment with simplified communities alters recovery of indigenous microbes. We used linear discriminant analysis effect size (LEfSe) to identify significantly enriched or depleted taxa between treatments that accelerated microbiome recovery in Fig. 5C (M-FMT, FS2C, and SC2 [Restored]) and treatments with more prolonged disruption (PBS, FS2A, and FS2B [Disrupted]). Independent analyses were performed for samples on days 1, 4 or 5, and 7 and relapse day 0; OTUs with LDA values determined by LEfSe of >3 for at least one time point are shown. Intensity of shading correlates to the log_2_-transformed median percent abundance measured for the treated mice at the indicated time points, with median abundances of >64% shaded dark blue and median values equal to 0.01% shaded dark yellow. Samples in which sequences were below the detection limit (ND, not detected) are shaded pale yellow. OTUs in bold typeface are indigenous to ^HMb^mice as described in Table S2. OTUs in standard typeface were found only in simplified communities. *Blautia* number 31 and *Bacteroides* number 3 were found in both ^HMb^mice and simplified communities. *Peptostreptococcaceae* number 35 is likely C. difficile, as the representative sequence has 100% identity to C. difficile and abundance over the course of infection correlates well with C. difficile levels reported in Fig. 3 and 4. The complete set of OTUs identified by LEfSe is provided in Table S4.

We identified 98 OTUs that were significantly enriched or depleted in treatments that accelerated microbiome recovery (FMT, FS2C, and SC2) compared to treatments with more prolonged disruption (PBS, FS2A, and FS2B) for at least one of the time points tested (Table S3). We focused on OTUs with the largest predicted effect sizes (linear discriminant analysis [LDA] > 3 [Fig. 6]). Three OTUs, *Erysipleotrichaceae* number 4, *Bifidobacterium* number 12, and *Bacteroidales* number 19, were significantly enriched in FS2C-, SC2-, and FMT-treated mice at all time points. *Erysipelotrichaceae* number 8, *Blautia* number 31, and *Clostridium* XIVa number 80 were enriched in FS2C-, SC2-, and FMT-treated mice on day 1, whereas several *Porphyromonadaceae* OTUs (numbers 6, 7, 10, 75, 39, and 48) as well as three *Firmicutes* OTUs (*Erysipelotrichaceae* number 17, *Lachnospiraceae* number 109, and *Olsenella* number 48) were enriched in FS2C-, SC2-, and FMT-treated mice at later time points. On day 1 of infection, *Peptostreptococcaceae* number 35 and *Enterococcus* number 28 were enriched in the feces of PBS-, FS2A-, and FS2B-treated mice, whereas *Bacteroides* number 3, *Bacteroides* number 9, and *Parabacteroides* number 1 were enriched during the later stages of infection. These results demonstrate that return toward the baseline microbiome configuration correlates with restoration of members of multiple phyla (*Bacteroidetes*, *Firmicutes*, and *Actinobacteria*), whereas continued disruption correlates with increased abundance of *Bacteroides* OTUs and a *Peptostreptococcaceae* OTU that is likely C. difficile.

10.1128/mSphere.00387-20.8 TABLE S4 Abundance of OTUs that differed significantly between treatments that restored microbiome diversity towards baseline (FMT, FS2C, and SC2) and those that remained disrupted (PBS, FS2A, and FS2B). Download Table S4, XLSX file, 0.04 MB. Copyright © 2020 Auchtung et al. 2020 Auchtung et al. This content is distributed under the terms of the Creative Commons Attribution 4.0 International license.

### Bacteria from simplified communities persist in the feces of treated mice.

**FIG 7:**
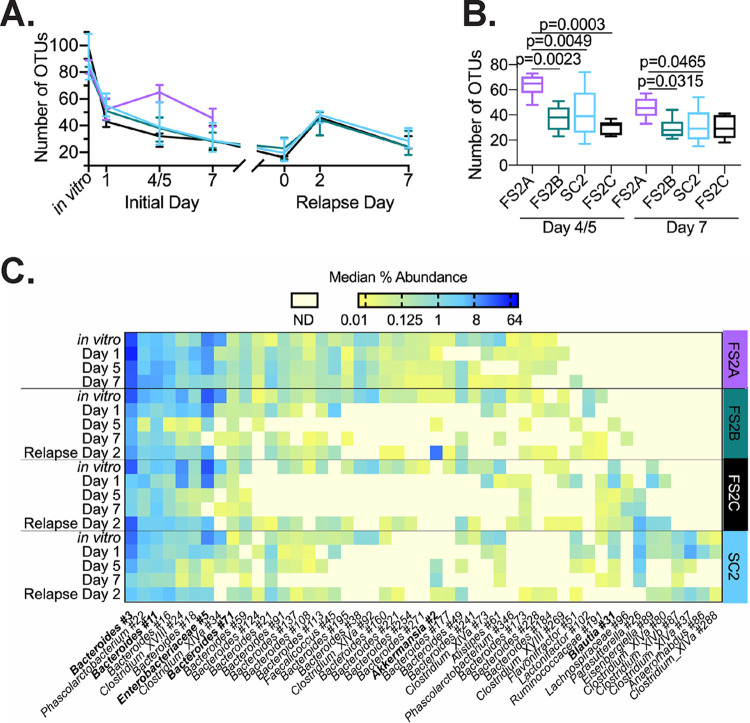
A subset of bacteria from simplified communities persists over time in treated mice. (A) Numbers of OTUs in SC2 (light blue), FS2A (violet), FS2B (teal), and FS2C (black) *in vitro* cultures that persist over time in the feces of treated mice. Lines represent median values at each time point, and error bars represent interquartile ranges. Significance of differences observed between treatment groups at each time point during the initial infection (days 1, 4 or 5, and 7) were evaluated with one-way Kruskal-Wallis testing with Dunn’s correction for multiple comparisons; *P* values of <0.05 are reported in panel B. (B) Data from days 4 or 5 and 7 replotted from panel A. The box represents the interquartile range, horizontal line indicates the median, and vertical lines indicate the range of data collected from fecal communities. (C) Log_2_-transformed percent abundance of OTUs that persist over time in mice treated with SC2, FS2A, FS2B, and/or FS2C. OTUs detected in *in vitro* samples were designated persistent if the median level in a treatment group on day 7 and/or relapse day 2 sample was >0.01%. Intensity of shading correlates to the median percent abundance measured for the treated mice at the indicated time points. OTUs in boldface type have also been detected in ^HMb^mice as described in Table S2. Abundance data from persistent OTUs at later times during infection (relapse days 0 and 7) and from OTUs abundant in day 0 samples that did not persist over time in treated mice are presented in Table S5.

We tracked the fate of OTUs present in *in vitro*-cultured simplified communities over time in mice treated with simplified communities. On day 1 following infection, ∼60% of OTUs present in *in vitro-*cultured simplified communities could be detected in the feces of treated mice (Fig. 7A; median OTU levels ranged from 43 to 55 OTUs). Levels of simplified community OTUs decreased over time, with the lowest number of OTUs (16 to 23) detected on relapse day 0 (Fig. 7A). Following induction of relapse, the number of OTUs detected from the original *in vitro*-cultured simplified communities increased to 44 to 48. These results indicate that these OTUs had likely persisted below the level of detection and reemerged when other OTUs declined following clindamycin treatment. OTUs that persisted over time were phylogenetically diverse (Fig. 7C). High levels of a *Phascolarctobacterium* OTU (number 22) and three *Bacteroides* OTUs (numbers 3, 11, and 16) were detected across all community-treated mice, indicating that these bacteria likely engrafted well.

10.1128/mSphere.00387-20.9 TABLE S5 Persistence of OTUs present in SC2, FS2A, FS2B, and FS2C over time in treated ^HMb^mice. Download Table S5, XLSX file, 0.03 MB. Copyright © 2020 Auchtung et al. 2020 Auchtung et al. This content is distributed under the terms of the Creative Commons Attribution 4.0 International license.

The trend for preservation of OTUs from simplified communities followed a different trajectory in FS2A-treated mice. The median number of FS2A OTUs detected increased to 65 on day 4 or 5 and returned to 46 on day 7 (Fig. 7A); values were significantly higher than those observed in mice treated with other simplified communities (Fig. 7B). Consistent with this observation, several *Bacteroides* OTUs present in *in vitro* cultures of all four simplified communities were only detected in the feces of FS2A-treated mice on day 7. This increased persistence of OTUs from FS2A was also consistent with the delayed return to baseline microbiome composition observed in these mice (Fig. 5C).

### Bacteria originating from simplified communities reemerge during relapse.

**FIG 8:**
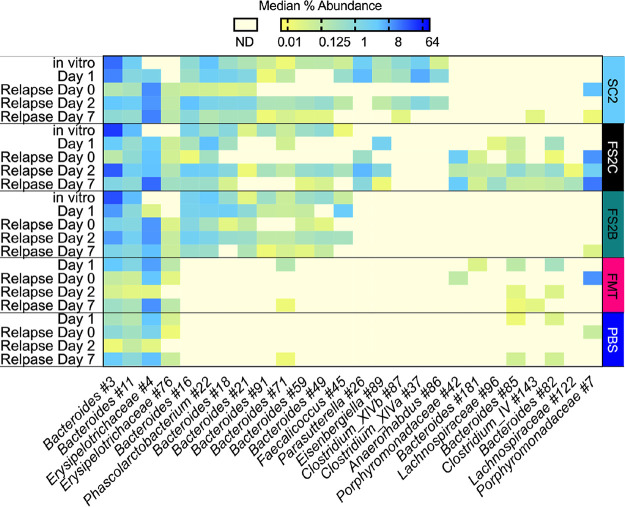
Treatment with SC2, FS2C and FS2B led to distinct microbiome responses during disease relapse. LEfSe was used to identify OTUs that differed significantly between treatment groups in the feces of mice on days 2 and 7 following induction of relapse with clindamycin i.p. Independent analyses were performed for samples on relapse days 2 and 7; OTUs with LDA values determined by LEfSe of >3 for at least one time point are shown. The complete set of LEfSe data is shown in Table S6. Median abundance of OTUs in *in vitro* SC2, FS2C, and FS2B cultures as well as all the feces of treated mice on day 1 and relapse day 0 samples are included for comparison as described in the text.

We also investigated significant differences in microbiome composition between treatment groups during relapse and identified 26 OTUs that showed high levels of difference between treatment groups during relapse. Approximately 70% of these OTUs enriched in the feces of SC2-, FS2C-, and FS2B-treated mice on relapse day 2 likely originated from the *in vitro*-cultured simplified communities (Fig. 8). Approximately 20% of OTUs enriched on relapse day 2 were enriched on day 1 of infection, indicating that the response to relapse was not identical to the initial disruption but shared some similarities. Similar to the kinetics observed during initial infection (Fig. 6), 11 of 26 OTUs were also significantly enriched on relapse day 7, while the remainder had decreased in abundance.

10.1128/mSphere.00387-20.10 TABLE S6 Abundance of OTUs in control and microbe-treated ^HMb^mice that differ significantly between treatments. Download Table S6, XLSX file, 0.08 MB. Copyright © 2020 Auchtung et al. 2020 Auchtung et al. This content is distributed under the terms of the Creative Commons Attribution 4.0 International license.

## DISCUSSION

We described a new pipeline for identifying and rigorously testing simplified communities with the ability to protect against C. difficile infection. We identified 24 new simplified communities with the ability to inhibit C. difficile *in vitro*. Several of the OTUs detected in these simplified communities were classified into families (*Lachnospiraceae*, *Ruminococcaceae*, *Clostridiaceae*, and *Bacteroidaceae*) and genera (*Bacteroides*, *Clostridium* XIVa, *Anaerostipes*, *Coprococcus*, *Dorea*, *Roseburia*, and *Blautia*) found depleted in the fecal microbiomes of people who are susceptible to C. difficile and restored following FMT treatment (43–47). In contrast, some OTUs were classified into families less often linked to resistance to C. difficile colonization (e.g., *Veillonella*, *Eggerthella*, *Clostridium* XVIII, and *Acidaminococcus*) or more correlated with susceptibility to C. difficile infection (*Enterococcus*, *Streptococcus*, and *Escherichia/Shigella*). Thus, the approach we described leads to communities distinct from those based upon predictive ecological modeling and may provide additional insights into C. difficile colonization resistance.

By testing simplified communities in an ^HMb^mouse model, we determined that only a subset of the tested communities conferred protection *in vivo.* Treatment with one community, SC4, resulted in loss of body mass consistent with toxicity of one or more members of this community. Although it is not clear which community member(s) in SC4 are toxic, SC4 contains a *Bilophila* OTU that could be the source of toxicity; treatment of specific-pathogen-free (SPF) mice with Bilophila wadsworthia caused weight loss as a result of systemic inflammation (48). Treatment with SC1, SC2, FS2B, and FS2C significantly reduced the initial body mass loss associated with severe disease and decreased C. difficile loads early in infection, similar to treatment with ^HMb^mouse FMT (M-FMT). While the magnitudes of effects varied, we observed a significant negative correlation between C. difficile levels on day 1 of infection and body mass on day 2 of infection (Fig. S4), with lower levels of C. difficile on day 1 predictive of reduced body mass loss on day 2 of infection. Thus, a potential mechanism for simplified communities to limit the severity of CDI *in vivo* is by delaying the germination or outgrowth of C. difficile spores. Similar reductions in C. difficile levels on day 1 following infection coupled to an ∼50% reduction in body mass loss were reported by Buffie et al. (22) when mice were treated with a consortium of four strains. In this case, early reductions in C. difficile levels were linked with restoration of secondary bile acid production by Clostridium scindens, as well as unknown functions contributed by other members of the simple community. Delaying germination or outgrowth could prevent severe disease by altering the dynamics of the host immune response between proinflammatory responses known to cause disease and anti-inflammatory responses that provide protection from C. difficile epithelial damage (21, 49, 50).

10.1128/mSphere.00387-20.4 FIG S4 Correlation analysis of levels of C. difficile and body mass change during initial infection. Correlation analysis of percent body mass on day 2 of infection relative to C. difficile levels on day 1 (A), day 4 or 5 (B), or (C) day 7 from all mice tested in experiments 1 to 3. Linear regression formulas and correlation coefficients for each plot are indicated in the corner of each graph. For regression plots, *P* < 0.0001. Download FIG S4, PDF file, 0.5 MB. Copyright © 2020 Auchtung et al. 2020 Auchtung et al. This content is distributed under the terms of the Creative Commons Attribution 4.0 International license.

Comparison of microbiome changes in the feces of mice treated with communities that limit (FS2C, SC2, and M-FMT), partially limit (FS2B), or fail to limit (FS2A and PBS) CDAD may explain some of the observed differences in disease progression. Treatment with FS2C, SC2, or M-FMT significantly limited body mass loss and altered the levels of C. difficile shedding; these communities also exhibited a more rapid return toward the baseline microbiome configuration observed in ^HMb^mice not treated with antibiotics. Return toward baseline was associated with an increased abundance of members of the indigenous microbiome, including several *Porphyromonadaceae* OTUs. *Porphyromonadaceae* were found to be depleted in the feces of humans and mice susceptible to C. difficile (51, 52). Enhanced restoration of indigenous microbes observed in FS2C- and SC2-treated mice could be due to the restoration of syntrophic interactions between indigenous microbes and those found in simplified communities and/or suppression of factors (i.e., C. difficile metabolism [53] and innate immune activation [54]) that promote microbiome disruption.

Mice treated with FS2B showed limited body mass loss during initial infection but did not show significantly altered C. difficile shedding in feces and exhibited a slower return to baseline microbiome conditions. A return to baseline microbiome conditions could, therefore, be important for C. difficile clearance but may not be required to mitigate initial disease severity. Treatment with FS2A, which failed to provide protection *in vivo*, was associated with significantly lower levels of restoration of indigenous microbes. Previous reports have indicated that specific probiotic formulations can delay the return to a nondisrupted microbiome configuration due to suppression of indigenous microbes (55); this could also be true for FS2A-treated mice. Further studies are needed to evaluate these hypotheses.

A subset of OTUs that originated from simplified communities persisted over time in the feces of treated mice. While the abundance of these OTUs diminished over time, continued colonization was demonstrated following induction of relapse. Persistence of these simplified community OTUs likely played a key role in limiting susceptibility to recurrent disease. One OTU of note was *Phascolarctobacterium*. A recent study demonstrated that administration of *Phascolarctobacterium* species to cefoperazone-treated mice reduces mortality, possibly by competing with C. difficile for succinate in the disrupted gastrointestinal (GI) tract (56). Other OTUs of note include those classified as *Blautia*, *Ruminococcaceae*, and *Eisenbergiella*. Colonization with members of the *Ruminococcaceae* family and the *Eisenbergiella* and *Blautia* genera was correlated with a 60% reduced risk for CDI in allogeneic hematopoieitic stem cell patients (57). Our results are also consistent with a previous study of microbiome restoration following FMT in human patients that found a balance between engraftment of donor bacteria, persistence of bacteria present in the feces of infected patients, and emergence of previously undetected bacteria (58).

Dilution-extinction provided a rapid way to screen simplified communities for the ability to prevent C. difficile infection. Development of diverse treatment consortia for CDI is important, as C. difficile is known to fill different nutritional niches (59) and fecal transplant studies indicate differential engraftment of species between patients treated with the same fecal sample (60). However, further refinement is needed before communities progress to clinical testing. Isolation of individual strains from simplified communities prior to community reassembly and efficacy testing will ensure the identity of the treatment consortia. In spite of these limitations, the approaches outlined in this report represent a significant advance in the throughput of testing for simplified communities to limit C. difficile infection and could potentially be adapted to identify simplified communities to treat other diseases linked to microbiome disruption.

## MATERIALS AND METHODS

### Fecal samples, bacterial strains, and cultivation conditions.

Fecal samples were provided by anonymous subjects between the ages of 25 and 64 years who self-identified as healthy and had not consumed antibiotics for at least 2 months or probiotics for at least 2 days prior to donation. Fecal samples were prepared as described previously (61). The previously described ribotype 027 isolate C. difficile 2015 was used for all experiments (61). All cultivation was performed at 37°C under an atmosphere of 5% H_2_, 5% CO_2_, and 90% N_2_.

### Identification of simplified communities through dilution/extinction.

Fecal samples were prepared and inoculated into MBRAs containing bioreactor medium 3 (BRM3) (62) as described previously (40). Briefly, frozen fecal aliquots were thawed and used to prepare 25% (wt/vol) fecal slurries in PBS under anaerobic conditions. After being vortexed for 5 min, the slurries were briefly centrifuged (5 min, 201 × *g*), and clarified supernatants were used to inoculate the reactors. Fecal communities were equilibrated for 16 h in batch growth before initiation of continuous flow (flow rate of 1.875 ml/h; 8-h retention time). After 5 to 6 days, an aliquot was removed for determination of cell concentration through serial dilution and plating on BRM3 agar and determination of C. difficile colonization status by PCR as described below. (Analysis was limited to D2 to D4 and D6 communities; D1 and D5 communities were not tested.) After 8 days, a sample was removed from each reactor and diluted to a final concentration of ∼3 × 10^4^ cells/ml (10^−4^ dilution) or 3 × 10^3^ cells/ml (10^−5^ dilution) in BRM3. One milliliter of each dilution was used to inoculate 5 or 6 sterile bioreactors containing 15 ml of sterile BRM3/dilution. After 3 days under continuous flow, aliquots were removed from diluted communities for sequencing and for cryopreservation with 15% glycerol or 7.5% dimethyl sulfoxide (DMSO). One day later, communities were challenged with 10^4^ C. difficile cells as described previously (61); C. difficile levels in reactors were determined through selective plating on TCCFA agar with 20 μg/ml of erythromycin and 50 μg/ml of rifampin as described previously (61). For repeat cultivation, cryopreserved stocks were thawed and 300 μl was used to inoculate triplicate reactor vessels containing 15 ml of sterile BRM3. Communities were grown in batch for 16 h and then with continuous flow for 4 days prior to challenge with C. difficile as described above.

### Analysis of C. difficile colonization status in undiluted communities D2, D3, D4, and D6.

As described above, 1-ml samples were removed from undiluted cultures on day 5 of cultivation. Cells were concentrated by centrifugation at ∼20,000 × *g* for 1 min. The supernatant was discarded and cells were lysed by bead-beating in sterile water as previously described (40). One microliter of cell lysate was used as the template in PCRs to detect C. difficile *tcdA* or bacterial 16S rRNA (broad-range 16S rRNA primers) using previously described primers (61) added at a final concentration of 1 μM to reaction mixtures containing Denville *Taq* master mix otherwise formulated according to the manufacturer’s instructions. After 35 cycles of amplification (94°C for 45 s, 55°C for 30 s, and 72°C for 1 min), 10-μl volumes of products were analyzed by agarose gel electrophoresis. Undiluted cultures had positive signals for the 16S rRNA gene as expected and no signal for C. difficile *tcdA*.

### Further simplification of SC1 and SC2.

One-milliliter stocks were thawed and used to inoculate an empty reactor vessel. The flow of sterile BRM3 was initiated and allowed to fill the reactor at a flow rate of 1.825 ml/h. After 3 days, cell concentrations were determined as described above. Two days later, aliquots of cells were removed and diluted in sterile BRM3 to a final concentration of 250 cells/ml (10^−6^ dilution) or 25 cells/ml (10^−7^ dilution). One-milliliter aliquots of cells were used to inoculate 5 (10^−6^ dilution) or 6 (10^−7^ dilution) empty, sterile reactors, which were allowed to fill with sterile medium as described above. After 2 (SC2) or 3 (SC1) days of flow, aliquots were removed for sequence analysis and cryopreservation. One (SC1) or 15 (SC2) days later, simplified communities were challenged with 10^4^ vegetatively growing C. difficile cells and levels of C. difficile persisting in reactors over time were determined through selective plating.

### Cultivation of simple communities for the treatment of ^HMb^mice.

Bioreactors (65 ml) were prepared as previously described (63). Sterile, empty bioreactors were inoculated with 1 ml of thawed stocks and allowed to fill with sterile BRM3 at a flow rate of 8.125 ml/h. Communities were cultured with flow for 6 to 8 days before 10-ml aliquots of culture were removed, centrifuged (800 × *g* for 10 min), and resuspended in 1-ml anaerobic PBS for delivery to mice. Cell densities of reactor communities were determined through selective plating on BRM3 agar; mice received doses ranging from 5 × 10^8^ to 2 × 10^9^ cells freshly prepared from reactors on three subsequent days.

### Preparation of ^HMb^mouse FMT and human FMT material.

Fecal samples were collected from 6- to 10 week-old male and female ^HMb^mice (42), pooled, and resuspended in anaerobic PBS at 20% (wt/vol). Samples were vortexed for 5 min and then centrifuged at 200 × *g* for 2 min. Each mouse was treated with 100 μl of clarified fecal slurry. The human FMT preparation was prepared as described previously (64).

### Treatment of ^HMb^mice with PBS, human FMT, ^HMb^mouse FMT, or simplified communities.

Antibiotics (61) were administered in the drinking water to 6- to 10-week-old male and female mice (Fig. 3A). Mice were treated with 100 μl of PBS, ^HMb^mouse FMT(M-FMT), human FMT (H-FMT), or cells from simplified communities via orogastric gavage on three subsequent days. Clindamycin (10 mg/kg of body weight) was administered via intraperitoneal injection. Mice were challenged with 5 × 10^4^ spores of C. difficile 2015. Three mouse experiments were performed. Mice in experiment 1 were treated with PBS, M-FMT, H-FMT, or SC1 to SC4 (*n* = 9 mice/treatment group except SC4 [*n* = 8]). Mice in experiment 2 were treated with PBS, M-FMT or SC2, FS2A, or FS2B (*n* = 9 mice/treatment group except PBS [*n* = 10]). Mice in experiment 3 were treated with PBS, M-FMT or SC2, FS2B, or FS2C (*n* = 8 mice/treatment group). In experiments 1 and 2, ∼100 μl of inoculum from the 3rd treatment was saved for sequencing. Mouse body mass was collected daily from days 0 to 5 following C. difficile challenge and then periodically following resolution of severe disease as indicated in figures. Mice that lost greater than 20% body mass from day 0 or showed signs of severe disease as previously described (42) were euthanized. Mouse body mass was also collected on day −2 and day −1 in experiment 1. Relapse was induced 24 (experiment 3), 28 (experiment 1), or 33 (experiment 2) days following initial C. difficile infection through i.p. administration of clindamycin (10 mg/kg). C. difficile levels in fecal samples were determined through selective plating (experiment 1) or qPCR (experiments 2 and 3) as described previously (61) at the time points indicated.

### Analysis of microbial communities through 16S rRNA gene sequencing.

Nucleic acids were extracted from mouse fecal samples and inoculum samples using the DNeasy Powersoil HTP kit (Qiagen) and from the further-simplified SC2 samples using the Powermag microbiome kit (MoBio). The V4 region of the 16S rRNA gene was amplified from purified DNA or directly from lysed bioreactor samples in triplicate using dual or single indexed primers F515/R806 as described previously (40, 65). Samples were cleaned quantified and pooled in equimolar concentrations prior to sequencing using the Illumina MiSeq v2 2 × 250 kit as described previously (40).

All sequence analysis was performed using mothur version 1.35.1. Raw sequencing reads were quality filtered, aligned to the V4 region of Silva 16S rRNA reference release 132, preclustered into sequence groups with <1% sequence divergence, filtered to remove chimeras with uchime, and classified with the Bayesian classifier using rdp database version 16 (>80% confidence threshold) as previously described, with the modifications noted above (40, 66). Sequence data were then rarefied to remove those sequences with <10 reads. Pairwise distance matrices were calculated and sequences were clustered into OTUs with >97 and >99% ANI. OTUs were classified by the majority consensus rdp taxonomy within the OTU. To better determine the potential identity of *Peptostreptococcaceae* OTU number 31 (>97% ANI) and *Peptostreptococcaceae* OTU number 35 (>99% ANI), representative sequences from these OTUs were compared to the nr/nt database using BLAST.

Samples were randomly subsampled to 10,000 sequences before determination of alpha and beta diversity measures. Alpha diversity measures (number of observed OTUs, inverse Simpson diversity, and Simpson evenness) were calculated using mothur. Principal-coordinate analysis of Bray-Curtis dissimilarities between communities were calculated and ordinates were visualized using the Phyloseq package (version 1.30.0 [67]) running in R version 3.61. Simplified communities were partitioned into four community types with Dirichlet multinomial mixtures as implemented in mothur version 1.44.0. Identification of OTUs significantly enriched between treatment groups in mouse experiments was performed using the mothur implementation of LEfSe (40, 66). mothur was also used to calculate the Bray-Curtis dissimilarities between treated mice and the baseline ^HMb^mouse sample (similarity = 1 − Bray-Curtis dissimilarity).

### Ethics statement.

Protocols for fecal sample collection were reviewed and approved by the Institutional Review Boards of Michigan State University and Baylor College of Medicine. Animal use was reviewed and approved by the Institutional Animal Care and Use Committee at Baylor College of Medicine (protocol number AN-6675).

### Data visualization and statistical analysis.

Unless otherwise noted, data were visualized and statistical analysis was performed using Prism v8.

### Data availability.

16S rRNA gene sequence data have been deposited in the Sequence Read Archive with accession number PRJNA632592.
