# Supplementary figures and images for: Identification of Simplified Microbial Communities That Inhibit Clostridioides difficile Infection through Dilution/Extinction
Source: mSphere. 2020 Jul 29;5(4):e00387-20. doi: 10.1128/mSphere.00387-20 (PMC7392540; doi:10.1128/mSphere.00387-20)

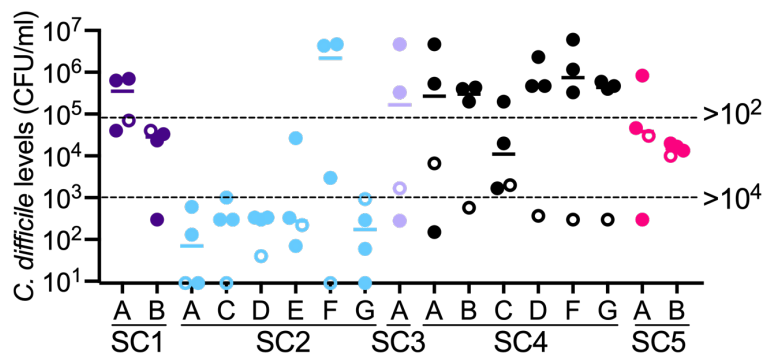

Figure S1

Supplement: FIG S1 [file mSphere.00387-20-sf001.pdf]

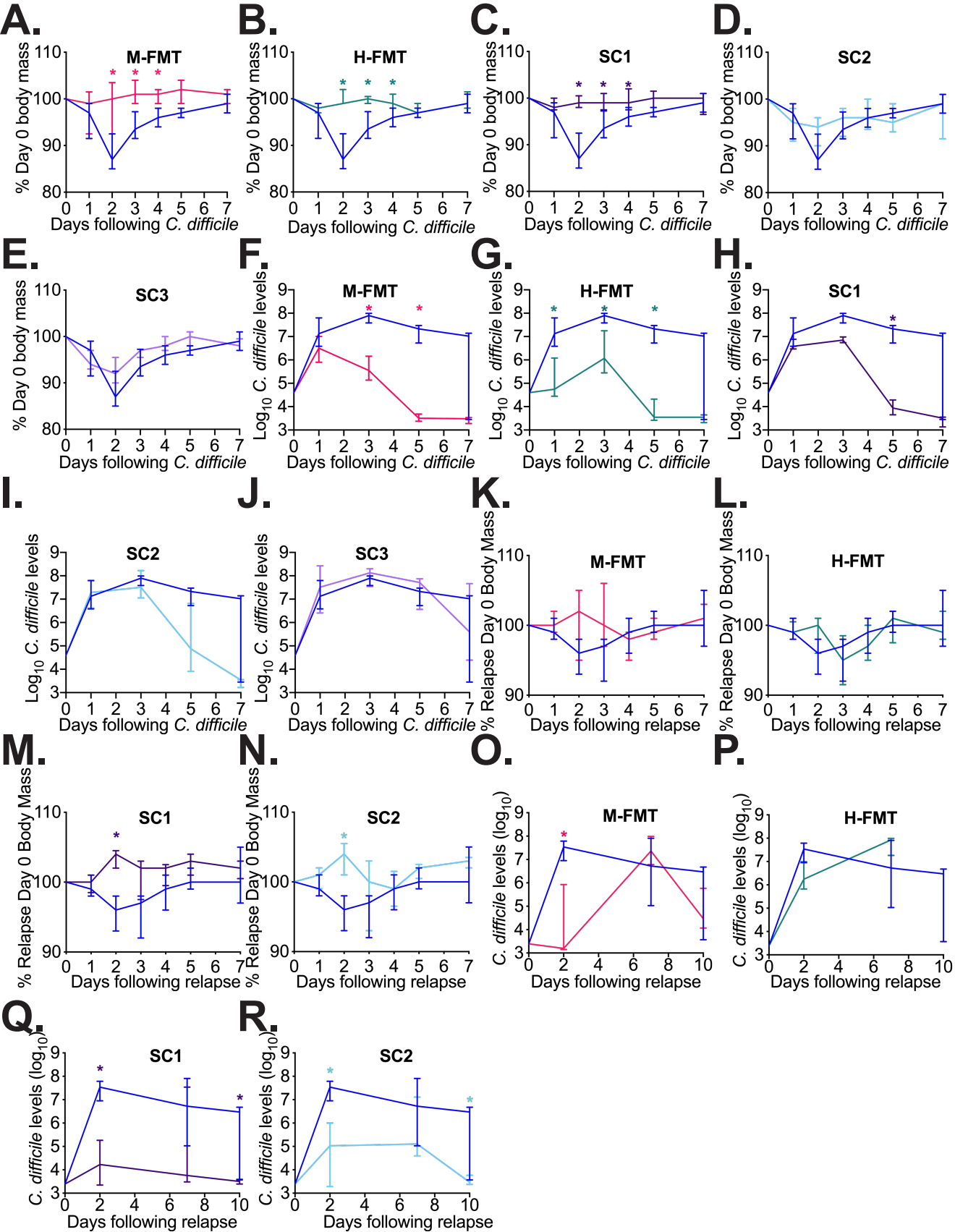

Figure S2

Supplement: FIG S2 [file mSphere.00387-20-sf002.pdf]

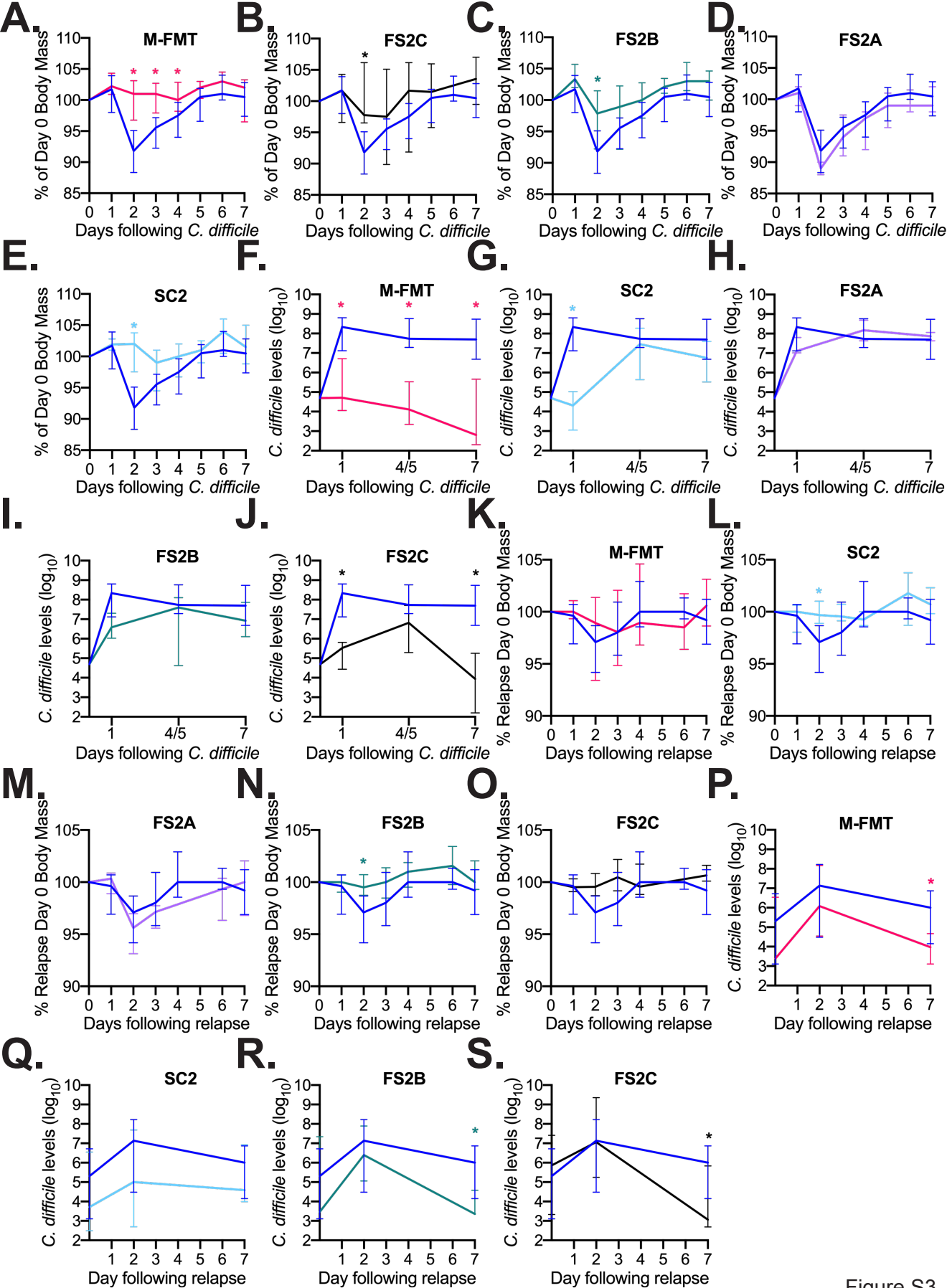

Figure S3

Supplement: FIG S3 [file mSphere.00387-20-sf003.pdf]

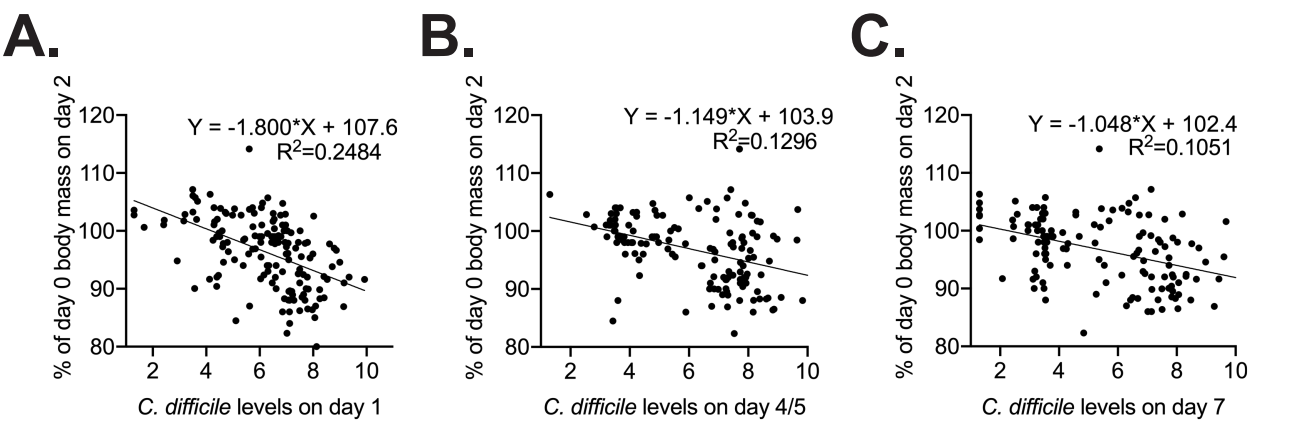

Figure S4

Supplement: FIG S4 [file mSphere.00387-20-sf004.pdf]
